# Socioeconomic Status and Prevalence of Obesity and Diabetes in a Mexican American Community, Cameron County, Texas, 2004-2007

**Published:** 2010-04-15

**Authors:** Susan P. Fisher-Hoch, Anne R. Rentfro, J. Gaines Wilson, Jennifer J. Salinas, Belinda M. Reininger, Blanca I. Restrepo, Joseph B. McCormick, Adriana Pérez, H. Shelton Brown, M. Monir Hossain, Mohammad H. Rahbar, Craig M Hanis

**Affiliations:** Division of Epidemiology, University of Texas School of Public Health, Brownsville Campus; University of Texas at Brownsville, Brownsville, Texas; University of Texas at Brownsville, Brownsville, Texas; University of Texas Health Science Center, Houston, School of Public Health, Brownsville campus, Brownsville, Texas; University of Texas Health Science Center, Houston, School of Public Health, Brownsville campus, Brownsville, Texas; University of Texas Health Science Center, Houston, School of Public Health, Brownsville campus, Brownsville, Texas; University of Texas Health Science Center, Houston, School of Public Health, Brownsville campus, Brownsville, Texas; University of Texas Health Science Center, Houston, School of Public Health, Austin campus, Austin, Texas; University of Texas Health Science Center, Houston, School of Public Health, Austin campus, Austin, Texas; University of Texas School of Public Health and The Center for Clinical and Translational Sciences at The University of Texas Health Science Center, Houston, Houston, Texas; University of Texas School of Public Health and The Center for Clinical and Translational Sciences at The University of Texas Health Science Center, Houston, Houston, Texas; University of Texas Health Science Center, Houston, School of Public Health, Houston campus, Houston, Texas

## Abstract

**Introduction:**

Mexican Americans are at increased risk for obesity and diabetes. We established a cohort on the United States-Mexico border to determine the prevalence of obesity and diabetes in this Mexican American population and to see whether minor economic advantages had any effect on health.

**Methods:**

We randomly selected and extensively documented 810 people aged 35 to 64 years. Weighted data were analyzed to establish prevalence of obesity and diabetes and other markers of poor health such as elevated glycated hemoglobin levels.

**Results:**

Rates of obesity (body mass index ≥30 kg/m^2^) were 57% in the first (lower) of 4 socioeconomic strata by income and were 55.5% in the third (higher). People in the higher socioeconomic stratum were significantly less likely to have undiagnosed diabetes (2% vs 9%). Among people aged 55 to 64 years, rates of diabetes were significantly higher among those in the lower socioeconomic stratum than among those in the higher stratum. Rates of undiagnosed diabetes had similar differences. Approximately three-fourths of the respondents reported having no health insurance, and we found no difference between people in different socioeconomic strata.

**Conclusion:**

Rates of obesity and diabetes in this border community are among the highest in the United States. Belonging to the lower socioeconomic stratum significantly increased the likelihood of having undiagnosed diabetes and, in patients too young to be eligible for Medicare, the overall risk of developing diabetes. Modest improvement in income has a beneficial effect on health in this racial/ethnic minority community.

## Introduction

Mexican Americans are at higher risk for obesity and diabetes than the general US population (1-5). The economic and social ramifications of these epidemics are substantial (6,7). In 2006, more than 20 million Americans were estimated to have type 2 diabetes, and nearly one-third of the cases were undiagnosed (8). By 2050, the number of US patients with diagnosed diabetes is projected to rise to 39 million (9). Race and ethnicity, encompassing genetics, culture, economics, and elements of the environment, are underlying risk factors for diabetes (9). In the United States, racial/ethnic minority populations, including Mexican Americans, have a higher risk of diabetes, but this tendency is confounded by socioeconomic status (SES) (10,11). Similarly, even though health in the United States, including diabetes control, improved in general from 1999 through 2006, this did not hold true in socioeconomically disadvantaged racial/ethnic minority populations, particularly before the age of eligibility for Medicare (≥65 years) (10,12).

Racial disparities measured in years of potential life lost from major diseases, including diabetes, are well documented (13). The influx of immigrants from Mexico has been in the forefront of a national debate over the past decade or more. What is often lost in this debate is the health status of Mexican Americans in the United States, and particularly on the United States-Mexico border where they are often the dominant ethnic group. Brownsville (Cameron County, Texas; 2006 population: 172,437 [14]) provides a unique environment to examine health disparities in Mexican Americans in the United States. This community provides an advance view of health outcomes due to demographic changes that are likely to spread to many parts of the country. In 2004, we began to recruit a cohort, now numbering 2,000, drawn from randomly selected households on the basis of 2000 Census tract data (15), with the purpose of establishing a "Framingham-like" cohort in this population. The Framingham cohort originated in 1948 as the Framingham Heart Study, consisting of a random sample of 5,209 adults from Framingham, Massachusetts, aged 30 to 62 years (16). This cohort has grown to serve numerous studies and supply risk factor data for common diseases. The primary objective of our study was to see what effect minor economic advantages might have on the prevalence of obesity and diabetes in a Mexican American population.

## Methods

### Study design

After early discussions with and observations in our community, we decided in 2004 to establish the Cameron County Hispanic Cohort (CCHC). In this study, we randomly selected a subset of people (aged 35-64 years) in CCHC cohort households to determine the influence of SES on diabetes and obesity. By using a 2-stage stratified sampling design, we generated data from 810 people from a total population of 41,199 (55% women, 90% Hispanic).

### Participants

From 2004 through 2007, we recruited 2,000 participants to the CCHC who are residents of Brownsville, Texas, which has 8 international crossing points to Matamoros, Mexico. We classified census tracts into 4 strata by income and targeted sampling on the first (lowest) and the third socioeconomic strata. For simplicity, we will call these "lower SES" and "higher SES." Median annual household income was $17,830 or less for the lower SES stratum and $24,067 to $31,747 for the higher SES stratum (15). The resultant sampling frame from 47 census tracts was 1,579 inhabited census blocks (with a population of 136,366). From the 476 clusters (a cluster is a distinct census block unit in a specific census tract) in the tracts in the lower SES stratum, we selected 42 clusters by simple random sampling. In a similar way, from the 294 tracts in the higher SES stratum we selected 38 clusters. We sampled 11 tracts in the 42 clusters in the lower SES stratum and 11 tracts in the 38 clusters in the higher SES stratum. We omitted 1 in the higher SES stratum because it contained predominantly "winter Texans," mostly retired winter visitors from the northern United States and Canada, most of whom are not Hispanic. Winter Texans typically cluster geographically and seasonally in rental housing complexes and parks for recreational vehicles (17). We invited all the households in the selected census blocks to participate in the study. Overall, 71% of households approached elected to participate, but more in the lower SES stratum did so (78% vs 63%, *P* = .03). We then selected 1 person randomly from each household to participate in this study.

The institutional review boards at the University of Texas Health Science Center and the University of Texas at Brownsville reviewed and approved the protocol. They also approved the informed consent forms, which included permission to collect and store de-identified data and specimens for this and other studies.

### Main outcome measures

Two nurses and 4 field workers trained in good clinical practices in accordance with National Institutes of Health requirements, all bilingual and bicultural, conducted the study in Spanish and English. We asked participants to fast for at least 10 hours overnight before their visit to our clinical research unit, located centrally at Valley Baptist Medical Center in Brownsville. After confirming the duration of their fast, we obtained blood for clinical analyses and DNA. We rescheduled participants who had not fasted. We measured participants' weight with their shoes removed by using a portable electronic scale and recorded the weight to the nearest 0.2 kg. Height was measured to the nearest 0.2 cm by using a stadiometer. We calculated body mass index (BMI) and determined waist circumference (visceral adiposity) at the level of the umbilicus, with participants in a standing position and breathing normally, to the nearest 0.2 cm. We measured blood pressure according to the protocol described in the National High Blood Pressure Education Program (18) and used the mean of measures 2 and 3 for analysis. We used a Glucostat analyzer (Model 27, YSI, Inc, Yellow Springs, Ohio) for blood glucose analysis. If we encountered unexpected results, such as elevated glucose in a participant not self-reporting diabetes, we asked the participant to return for a second visit to confirm the result. We discussed results with each participant. When we found abnormal results, we referred the participant to his physician or, if he had none, to a local federally qualified health center clinic. We froze whole blood for hemoglobin A1c (HbA1c) analysis (19) and transported white cells and plasma on ice to be archived for further laboratory analyses and future studies. We used enzyme-linked immunosorbent assays to determine insulin levels (Mercodia, Uppsala, Sweden).

### Statistical analysis

We report results at the participant level. In the final sample, all participants were Hispanic, and 68% were female. This imbalance in the sample-generating mechanism needed to be adjusted in the analysis in order to generalize the results (eg, irrespective of sex), which we did by incorporating the sampling weights into our analysis. The sampling weights are the inverse probability of selection of each participant based on his or her SES stratum, census tract and block (cluster) of residence, and sex. Incorporating these sampling weights provides the correct statistical inference by giving a better estimate of standard error, and hence the confidence interval. In the sampling weight calculation, we made a post-ratio adjustment for the 2000 census population data. We also examined the data by ranking participants by household income and selecting the top and bottom quartiles to obtain a wider difference in household income than that provided by use of the census data. Thus, we compared the top 202 participants with the bottom 202 participants on the basis of household income.

We report results for the dependent variables of reported medical history and observed variables, including BMI, waist circumference, and fasting blood glucose and insulin. We used the homeostasis model assessment equation to determine insulin resistance (HOMA-IR = glucose (mg/dL)/18 × insulin (mU/L)/22.5) (20). Demographic covariates included age, sex, country of birth, country of birth of parents and grandparents, education, and employment status. First, we generated demographic and descriptive data to describe the sample. We compared weighted and unweighted analysis and determined that using the sample weights for the analysis was preferable, since they corrected for the lower response rate for men and the unemployed. We then developed a multiple logistic regression model to allow for the effects of potential confounders such as age, sex, and country of birth. Finally, we ranked the entire cohort by annual household income and repeated the analyses by using the bottom and top quartiles to see whether this would allow us to observe more subtle differences. We used SAS version 9.1 (SAS Institute, Inc, Cary, North Carolina) and Stata 10 SE (StataCorp LP, College Station, Texas). We made adjustments in the respective statistical packages for sampling design.

We used a geographic information system to visualize the spatial distribution of households by income quartile and the density of sampling. We geocoded households by using latitude and longitude coordinates collected by global positioning system, and crosschecked with the street addresses to ensure accuracy. We created maps and layouts with Environmental Systems Research Institute ArcMap v. 8.3 software (ESRI, Redlands, California).

## Results

**Figure1.**
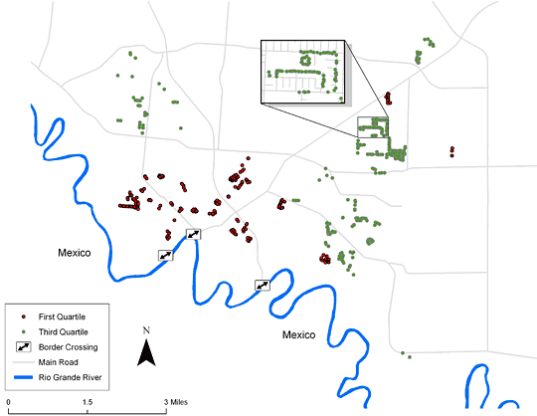
Map of Brownsville, Texas, derived using geographic information systems and showing the distribution of the cohort across the city, Cameron County Hispanic Cohort, 2004-2007. People in the first quartile had annual household incomes of $17,830 or less, and people in the third quartile had annual household incomes of $24,067-$31,747. The inset box shows detail of 1 of the blocks randomly sampled.

Participation in the cohort in Brownsville did not significantly differ by SES stratum (Figure 1 and Table 1). The only substantial difference between the 2 socioeconomic strata was the proportion of participants who completed high school. Less than a fourth of the participants reported having private health insurance. A few (5%) were on Medicaid and a few others (5%) reported having Medicaid or Medicare combined with private managed care.

More than half of the participants in both SES strata had BMI scores in the obese range (≥30 kg/m^2^) (Table 2). Slightly more participants in the higher SES stratum were morbidly obese (BMI ≥40 kg/m^2^), but the difference was not significant. Self-reported diabetes differed only slightly between strata, but we found significant differences between strata for undiagnosed diabetes. Nearly 1 in 10 of all participants in the lower SES stratum were informed through this study that they have diabetes. In both strata, mean HbA1c levels among participants with diabetes were 9.4%. In logistic regression models allowing for potential confounding variables, undiagnosed diabetes continued to be the only significant outcome associated with socioeconomic strata (Table 2).

The only significant difference in biological measurements by SES strata when using weighted data was for fasting blood insulin (Table 3). Results stratified by sex show significant differences between SES strata in women for fasting insulin and insulin resistance. Still, mean insulin resistance for both sexes was well above the upper limit of the normal range (3.15).

When we ranked these participants by annual household income, we found the mean household income of the fourth quartile to be $20,532 and of the first quartile to be $8,160. When we compared these quartiles, we found no differences in frequency of obesity or in mean BMI. However, consistent with what we observed using census tract quartiles, we found a significantly higher mean fasting blood glucose of 123.2 mg/dL (95% CI, 114.5-131.9 mg/dL; n = 204) in the first quartile compared with 108.0 mg/dL (95% CI, 101.3-114.5 mg/dL; n = 202) in the fourth quartile. We also observed a marked difference in prevalence of diabetes (diagnosed and undiagnosed), 21% (43/204) in the first quartile compared with 10% (20/202) in the fourth quartile (χ^2^ = 6.596, *P* < .01). We found no difference in mean insulin or insulin resistance levels between the 2 quartiles.

**Figure 2.**
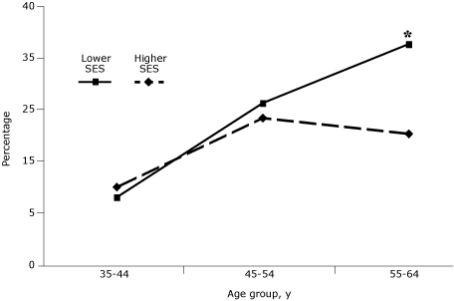
Percentage of participants with diabetes, by age and by socioeconomic status, Cameron County Hispanic Cohort, 2004-2007. People in the first quartile had annual household incomes of $17,830 or less, and people in the third quartile had annual household incomes of $24,067-$31,747. Among people aged 55 to 64 years, the difference between people in the lower socioeconomic stratum compared with those in the higher stratum was significant at* P* < .05.

Age had a marked influence on the effect of SES. Rates of diabetes were significantly higher for people aged 55 to 64 years in the lower SES stratum compared with those in the higher SES stratum (Figure 2). There was a decrease in self-reported diabetes and an increase in undiagnosed diabetes, which was also age-dependent.

**Table:** 

**Age group, y **	**Lower SES**	**Higher SES**
35-44	8	10
45-54	26	23
55-64	37	20

## Discussion

In a largely uninsured racial/ethnic minority population on the urban United States-Mexico border, SES was associated with undiagnosed diabetes and development of diabetes. Despite the narrow range of income in this population, people in the lower SES stratum were significantly more likely to have undiagnosed diabetes and the risk of diabetes increased markedly with age. This adverse effect of lower SES stratum on health became starker when we compared the top and bottom quartiles of self-reported household incomes. However, we found no differences between strata in prevalence of obesity.

We found that women in the lower SES stratum were more likely to have elevated insulin resistance and fasting insulin levels compared with women in the higher SES stratum, but we did not observe similar differences for men, consistent with published observations (21,22). Mexican American women in the lower SES stratum may have less access to health care than do their counterparts in the higher SES stratum, or perhaps lifestyle differences between strata contribute to how women care for their own health.

National data may underestimate the prevalence of obesity and diabetes in border communities (1). Rates of diabetes in these communities were 1.4 times higher than those reported nationally for Mexican Americans aged 20 years or older (1) and nearly twice as high as in all Americans (aged 40-59 years) (1,3,23). Undiagnosed diabetes was also twice as high in our border population as nationally. Similarly, our rates of obesity were more than one and a half times the rate in Mexican Americans nationally (aged 40-59 years), and one-tenth of our women were morbidly obese (3). These excess rates offer a glimpse of the potential future health of people of Mexican origin residing in other parts of the United States.

Our study sample is not yet eligible for Medicare (age ≥65 years). In both SES strata, nearly 80% of participants reported that they had no medical coverage. These data highlight the need for prevention and intervention aimed at people aged 35 or older but younger than 65, who are most at risk and least likely to be able to afford health care. This population is at particular risk for expensive complications such as renal failure and amputations. Complications of diabetes reduce ability to work, exacerbating the socioeconomic vulnerability of this age group.

There were several limitations to our study. Despite intensive efforts, more women than men participated in the study; this was not unexpected in a community where most men are on hourly wages. We could not assess those randomly selected people who chose not to participate. Exclusion of the second and fourth SES strata weakens our estimates of prevalence. Our data raise issues of acculturation and immigration experience, but we did not collect data to distinguish those factors and SES. Though the population is largely Mexican in origin, participants in this study had lived in Brownsville an average of 16 years, making them long-term residents of the United States. Nevertheless, more than 70% elected to answer the questionnaires in Spanish. Residents in Brownsville access Mexican culture freely through media outlets and imported products in local retail and grocery stores; those who can also move freely across the border. In many ways, Brownsville more closely resembles its Mexican neighboring city, Matamoros, than its closest major US city, Corpus Christi. The process of acculturation to Brownsville may differ from that observed in the general US population. We plan to gather precise acculturation data in our future studies with this cohort to determine patterns in this community.

The strengths of our study are considerable in that we have recruited the first exclusively Mexican American cohort in a border city with extreme health disparities. With the support of our clinical research unit funded by the National Institutes of Health, we have been able to recruit and process participants in a standardized fashion, by using staff expert in good clinical practices and by using well-monitored data management and analysis processes. This enables us to report reliable data that can be used to plan for appropriate prevention and equitable health care for an underserved population.

The overall picture of health in this Mexican American population is poor. The rate of diabetes in Hispanics/Latinos is nearly twice that in whites, occurring at an earlier age and with higher rates of complications and death (2). In Mexican Americans on the United States-Mexico border, published rates of diabetes in rural communities are more than twice those in non-Hispanic whites (24). We show the same elevated risk in urban border populations. Furthermore, we demonstrate high rates of diabetes, particularly undiagnosed diabetes, in the poorest of our poor who have little or no access to health care through insurance. This presents a major economic burden on the community and on the health care system because it delays diagnosis and treatment until the disease is far more complex and costly to address. Intervention in the form of community-based participatory research will likely be essential to achieve sustainable solutions (25). A focus on early diagnosis and prevention of diabetes will be key to success in border health.

## Figures and Tables

**Table 1:** Description of 810^a^ People Aged 35-64 Years, by SES^b^, Cameron County Hispanic Cohort, Brownsville, Texas, 2004-2007

Characteristic	Lower SES^c^	Higher SES^c^	*P* Value^d^
**Participation, n (%)**	350 (74.0)	460 (76.5)	NA
**Sex, n (%)**			
Women	232 (55.7)	319 (57.1)	.04
Men	118 (44.3)	141 (42.9)	
**Place of birth, n (%)**			
Mexico	239 (71.1)	338 (74.4)	<.001
United States	105 (28.9)	119 (25.6)	
**Place of birth for both parents, n (%)**			
Mexico	234 (88.3)	318 (87.7)	.19
United States	38 (11.7)	43 (12.3)	
**Place of birth for all 4 grandparents, n (%)**			
Mexico	238 (95.5)	328 (94.6)	.01
United States	12 (4.5)	14 (5.4)	
**Completed high school, n (%)**			
Yes	134 (35.3)	196 (45.3)	<.001
No	214 (64.7)	264 (54.7)	
**Employed, n (%)**			
Yes	85 (60.5)	107 (59.0)	.02
No	33 (39.5)	34 (41.0)	
**Health insurance, n (%)**			
Yes	91 (22.3)	92 (21.8)	<.08
No	259 (77.7)	368 (78.7)	
**Age, mean (SD)**			
Women	49.1 (0.81)	48.9 (0.87)	<.001
Men	47.6 (1.16)	48.4 (0.92)	<.001

Abbreviations: SES, socioeconomic status; NA, not applicable; SD, standard deviation.

a Numbers may not total 810 because of missing data.

b People in the first quartile had annual household incomes of $17,830 or less (lower SES), and people in the third quartile had annual household incomes of $24,067-$31,747 (higher SES).

c Unweighted frequencies.

d χ^2^ Tests and percentages using weighted data.

**Table 2:** Prevalence by SES Quartile^a^ and Odds Ratios from Logistic Regression Analysis^b^ of Obesity and Diabetes, Cameron County Hispanic Cohort, Brownsville, Texas, 2004-2007

**Characteristic**	Prevalence		Odds Ratio	

	Lower SES (N = 350), n (%)^c^	Higher SES (N = 460), n (%)^c^	Univariate (95% CI)	Multivariate^d^ (95% CI)
Obese (BMI ≥30)	189 (56.9)	253 (55.5)	0.97 (0.80-1.18)	1.00 (0.81-1.22)
Morbidly obese (BMI ≥40)	34 (7.7)	39 (8.3)	1.04 (0.82-1.32)	1.04 (0.79-1.28)
Self-reported diabetes^e^	50 (14.0)	66 (15.1)	1.03 (0.67-1.58)	1.07 (0.73-1.59)
Undiagnosed diabetes^f^	23 (8.8)	16 (2.5)	0.55 (0.33-0.93)	0.54 (0.32-0.93)^g^

Abbreviations: SES, socioeconomic status; CI, confidence interval; BMI, body mass index.

a People in the first quartile had annual household incomes of $17,830 or less (lower SES), and people in the third quartile had annual household incomes of $24,067-$31,747 (higher SES).

b Reference group is higher SES.

c Unweighted frequencies and weighted percentages.

d Controlling for age, sex, country of birth, education, and employment status.

e Respondents answered yes to the question "Has a doctor ever told you you have diabetes?"

f Respondents answered no to the question "Has a doctor ever told you you have diabetes?" but had fasting blood glucose on 2 occasions ≥126 mg/dL.

g Significant at* P* < .05.

**Table 3:** Estimated Means of Anthropometric and Biological Variables Using Weighted Data, by SES Quartile^a^, Cameron County Hispanic Cohort, Brownsville, Texas, 2004-2007

**Characteristic**	Lower SES (SD)	Higher SES (SD)	Mean Difference (95% CI)
**Body mass index (kg/m^2^)**	31.7 (0.49)	31.4 (0.33)	0.26 (−0.91 to 1.43)
Women	32.8 (0.65)	32.3 (0.42)	0.50 (−1.02 to 2.03)
Men	30.3 (0.6)	30.3 (0.43)	0.02 (−1.42 to 1.47)
**Waist circumference, cm**	103.2 (1.02)	101.9 (0.90)	1.34 (−1.32 to 4.00)
Women	102.2 (1.19)	100.5 (1.18)	1.67 (−1.62 to 4.95)
Men	104.5 (1.48)	103.7 (0.97)	0.84 (−2.62 to 4.30)
**Fasting blood glucose, mg/dL**	118.1 (4.47)	112.5 (2.88)	5.61 (−4.82 to 16.03)
Women	112.3 (3.34)	111.2 (3.45)	1.11 (−8.30 to 10.53)
Men	125.5 (8.87)	114.3 (4.59)	11.16 (−8.41 to 30.73)
**Fasting insulin, mU/L**	16.4 (1.05)	13.6 (0.45)	2.78 (0.55 to 5.01)^b^
Women	17.5 (1.0)	14.1 (0.43)	3.45 (1.32 to 5.58)^b^
Men	15.0 (1.52)	13.0 (1.02)	1.99 (−1.59 to 5.57)
**Insulin resistance ≤3.15**	4.8 (0.44)	3.8 (0.20)	0.92 (−0.03 to 1.87)
Women	5.0 (0.44)	4.0 (0.24)	0.99 (0.01 to 1.97)^b^
Men	4.5 (0.54)	3.6 (0.38)	0.86 (−0.45 to 2.16)
**Systolic blood pressure, mm/Hg**	120.7 (1.54)	119.8 (0.67)	0.85 (−2.43 to 4.13)
Women	118.9 (1.49)	118.4 (0.96)	0.53 (−2.94 to 3.99)
Men	122.8 (2.45)	121.7 (1.32)	1.15 (−4.31 to 6.61)
**Diastolic blood pressure, mm/Hg**	73.6 (1.02)	73.7 (0.53)	−0.07 (−2.33 to 2.19)
Women	71.7 (0.93)	71.6 (0.53)	0.12 (−1.99 to 2.22)
Men	76.1 (1.89)	76.6 (0.77)	−0.46 (−4.46 to 3.54)

Abbreviation: SES, socioeconomic status; SD, standard deviation; CI, confidence interval.

a People in the first quartile had annual household incomes of $17,830 or less (lower SES), and people in the third quartile had annual household incomes of $24,067-$31,747 (higher SES).

b Significant at at* P* < .05.
